# Turning the tide on inequity through systematic equity action-analysis

**DOI:** 10.1186/s12889-023-15709-5

**Published:** 2023-05-15

**Authors:** Katrina M. Plamondon, Jenna Dixon, Ben Brisbois, Rodrigo Curty Pereira, Elijah Bisung, Susan J. Elliott, Ian D. Graham, Sume Ndumbe-Eyoh, Stephanie Nixon, Sana Shahram

**Affiliations:** 1 Science of Health Equity Learning Lab & Assistant Professor, School of Nursing, University of British Columbia ART360, 1147 Research Road, Kelowna, BC Canada; 2 Science of Health Equity Learning Lab School of Nursing, University of British Columbia, Kelowna, BC Canada; 3 Science of Health Equity Learning Lab at the School of Nursing, School of Health Sciences, University of British Columbia, University of Northern British Columbia, Kelowna, Prince George, BC, BC Canada; 4 Geography and Environmental Management, University of Waterloo, Ontario, Canada; 5 School of Kinesiology & Health Studies, Queens University, Kingston, Canada; 6 Centre for Practice-Changing Research, School of Epidemiology & Public Health, Faculty of Medicine, Ottawa Hospital Research Institute, University of Ottawa, Ottawa, Canada; 7 Dalla Lana School of Public Health, Black Health Education Collaborative &, University of Toronto, Toronto, Canada; 8 Health Sciences & Director, School of Rehabilitation Therapy, Queens University, Kingston, Canada; 9 Science of Health Equity Learning Lab & Assistant Professor, School of Nursing, University of British Columbia, Columbia, Canada

**Keywords:** Health equity, Health inequities, Knowledge-to-action, Research methods, Tools, Practices, Equity action

## Abstract

**Background:**

Collective agreement about the importance of centering equity in health research, practice, and policy is growing. Yet, responsibility for advancing equity is often situated as belonging to a vague group of ‘others’, or delegated to the leadership of ‘equity-seeking’ or ‘equity-deserving’ groups who are tasked to lead systems transformation while simultaneously navigating the violence and harms of oppression within those same systems. Equity efforts also often overlook the breadth of equity scholarship. Harnessing the potential of current interests in advancing equity requires systematic, evidence-guided, theoretically rigorous ways for people to embrace their own agency and influence over the systems in which they are situated. ln this article, we introduce and describe the Systematic Equity Action-Analysis (SEA) Framework as a tool that translates equity scholarship and evidence into a structured process that leaders, teams, and communities can use to advance equity in their own settings.

**Methods:**

This framework was derived through a dialogic, critically reflective and scholarly process of integrating methodological insights garnered over years of equity-centred research and practice. Each author, in a variety of ways, brought engaged equity perspectives to the dialogue, bringing practical and lived experience to conversation and writing. Our scholarly dialogue was grounded in critical and relational lenses, and involved synthesis of theory and practice from a broad range of applications and cases.

**Results:**

The SEA Framework balances practices of agency, humility, critically reflective dialogue, and systems thinking. The framework guides users through four elements of analysis (worldview, coherence, potential, and accountability) to systematically interrogate how and where equity is integrated in a setting or object of action-analysis. Because equity issues are present in virtually all aspects of society, the kinds of ‘things’ the framework could be applied to is only limited by the imagination of its users. It can inform retrospective or prospective work, by groups external to a policy or practice setting (e.g., using public documents to assess a research funding policy landscape); or internal to a system, policy, or practice setting (e.g., faculty engaging in a critically reflective examination of equity in the undergraduate program they deliver).

**Conclusions:**

While not a panacea, this unique contribution to the science of health equity equips people to explicitly recognize and interrupt their own entanglements in the intersecting systems of oppression and injustice that produce and uphold inequities.

## Background

Inspired by the possibilities dwelling within each person, as members of the organizations, systems, and societies humans collectively construct, the late bell hooks invited people to use their imaginations for more equitable futures through joy, justice, and liberation. She invited people to choose love as a movement against oppression, enacting their own their agency in the world and embracing the possibility that, as the *essence* of systems, peoples’ hearts and minds shape the world [1]. hook’s invitation is a reminder of capabilities, humility, and choice in the context of systems that seem overwhelmingly outside of our control, and tenaciously designed to uphold imbalances in power.

Power and its role within organizations, systems, and societies can be understood by using health and health outcomes as windows to reveal something important about its distribution. Health outcomes serve as a measurable indicator of the health of society, and all of the systems, organizations, and groups within. Evidence identifies the distribution of resources, wealth, and power as causes of health inequities [2]; yet, despite decades of international proclamations of commitment to respond, they persist and were worsened by the COVID-19 pandemic [3]. Across research, policy and practice, broadly, there remains a tendency to: focus on downstream outcomes (symptoms) rather than upstream causes [4]; naturalize and minimize the complexity of health and social inequities, often by amplifying a focus on individuals and behaviours [5]; and rationalize extractive, exploitative power relationships couched in common biomedical narratives of benevolence [6]. Indeed, the entire health research enterprise cannot be divorced from the neocolonial, racist, eco-cidal, and patriarchal systems [7–9] that advance ideologies of biomedicine and capitalism, such as individualism and its entrenchment through contemporary ‘neoliberal’ capitalist transformations [10]. The impact of these dominances is a preoccupation with solutions that distract from, rather than respond to, known drivers of inequities.

In Canada, where we, as authors, are located, recognition of the importance of advancing equity through health research is growing. In 2021, the Canadian Institutes for Health Research (CIHR) joined other international funding bodies and research organizations in integrating evidence about health and social inequities into their own strategic policies. CIHR’s most recent strategic plan envisions a world where “social factors such as postal codes are no longer significant predictors of life and health expectancy,” wherein Canada acts as a “global leader in the science of achieving health equity” [11]. While these and other calls to centre equity in health research are promising, there remains limited collective capacity to understand and act on systems, policies, norms, and practices that (re)produce inequity.

Institutional efforts to produce ‘equity, diversity, and inclusion’ statements or establish ‘equity offices’ present a pivotal moment for shaping new practices and norms. Yet, many efforts remain performative with little to do with justice or liberation. Superficial engagement in equity work risks perpetuating hegemonies, using good intentions to reinforce structures and systems that produce inequities. Practical traps that prop this risk up tend to avoid the rigour of equity science, relinquishing equity work to ‘soft’ or atheoretical practices [12]. For example, frameworks narrowly focused on processes without sufficient critical analysis can distract attention away from causes of inequities. Critical analyses of some vague notion of distant and detached ‘systems’ or ‘structures’ can separate people from their influence over these systems. Both tend to situate the responsibility of advancing equity with others, separate from one’s own actions or day-to-day work. Further, much of the relational, procedural, administrative and leadership work is left to ‘equity-seeking’ or ‘equity-deserving’ groups who are tasked to lead systems transformation while simultaneously navigating the violence and harms of oppression within those same systems [13]. People can also get stuck in hopelessness, overwhelmed by the scope and scale of transforming political ideologies or power structures. Each of these traps can lead to superficial responses that uphold inequities. Harnessing the full potential of current interests in advancing equity requires systematic, evidence-guided, theoretically rigorous and practical ways of applying this scholarship.

ln this article, we introduce and describe the Systematic Equity Action (SEA) Framework as a tool for analysis and planned action. First, we offer a description of the methodological and theoretical foundations underpinning its development, situating the framework in the context of equity scholarship in population and public health. We then describe the conceptual and procedural elements of the SEA Framework. Finally, we show how this framework can be applied in different settings to guide tangible integration of equity practices and transformations across contexts. We provide example real-world applications of the framework in distinct administrative and organizational settings, demonstrating its utility as a systematic, practical process that leaders, teams, and communities can use to take up the collective work of equity-oriented systems transformations from a position of solidarity and learning. Our closing reflections invite people, across a variety of contexts within and beyond health-related settings, to embrace their essential role and agency in making equity choices within the systems they themselves constitute.

## Dialogic, critically reflective, evidence-informed foundations

The SEA Framework was derived through a dialogic and scholarly process of critical reflection among the named authors, integrating theoretical considerations alongside methodological and procedural insights garnered over years of applying equity-centred research and practice. All authors share an interest in the relationship between knowledge and action (e.g., Graham’s work on integrated knowledge translation), and between research and society. Our dialogue was grounded in critical and relational theoretical lenses [14, 15], and involved synthesis of theory and lessons learned through a wide range of studies, projects, and efforts to integrate equity considerations in systems settings. Our team brought a diversity of disciplinary perspectives and applied equity scholarship, including a balance of lived experiences among people navigating intersecting inequities and/or critical allyship (some of us navigate both). All of us brought experiences of engaging in equity work to conversation and writing. Our authorship team originally convened through a policy analysis project (CIHR Grant 451,833; Plamondon, PI; Elliott, Graham, Nixon Co-PIs; Dixon Research Associate; Curty Pereira Research Assistant; Shahram, Bisung, Ndumbe-Eyoh scholarly contributors), where we spent a portion of each team meeting in reflective dialogue about how the analytical process was serving our equity purposes. Seeds for this SEA Framework evolved from collaboration on other research efforts, including the elaboration of equity-centred principles to guide global health research (Plamondon, Nixon, and Brisbois were part of the original research team; Bisung, Elliott and Graham worked with these principles later). Plamondon’s doctoral research extended these aspirational questions into practice, focusing on questions of *how* to do equity-centred research and knowledge translation. Brisbois and Plamondon applied equity-centred critical analysis to the construction of worldview in global health [16]. Nixon’s work on privilege and allyship [17] was also influential, as were Shahram and Ndumbe-Eyoh’s work on building capacity for equity in health systems and academic settings [12, 18–20]. While our research experience demonstrated that advancing equity was *not* common sense or easy, it was enabled by taking incremental and systematic steps toward equity thinking and action. Early approaches to this framework were designed to overcome conceptual and practical traps we frequently encountered in our equity work [12]. Over two years of connecting to share our insights and reflections, we began to write about our approaches.

### Theoretical foundations & underlying assumptions

The SEA Framework is grounded in relational theory [14, 21, 22] and applied critical, anti-oppressive approaches in health science and practice [23–26]. It positions health and well-being as relevant windows through which we can understand broader social systems and structures; wherein the health and life trajectories of people (individuals, groups, communities, populations) are always situated in social and structural determinants of health [2]. Further, it is grounded in the notion that society is made up of people whose often un-recognized assumptions and power dynamics create and enforce systemic advantages and disadvantages. While we recognize that all people navigate these dynamics from intersecting positions, we acknowledge that some people experience far more disadvantage than others.

We understand health, health equity, and health inequities as relational constructs, meaning they are understood as existing in relationship to systems, structures, and social climates that are, in turn, shaped by relationships that exist between people, ideas, organizations, bodies of knowledge, and contexts [27]. Many cultures and knowledge systems embrace collectivism and interconnectedness. These knowledge systems sit in contrast to the white-euro-centric, linear-reductionistic, patriarchal, colonial, capitalist, and biomedicalized assumptions that dominate much of the health sciences. Critical theorists like Paulo Freire and bell hooks, and Indigenous scholars and knowledge-keepers such as Linda Tuhiwai Smith and Willie Ermine (among many others) offer wisdom with grace and generosity, inviting hopeful attentiveness to the systems and structures that hold society’s contradictions and injustices together. Common among these thought-leaders and knowledge keepers is recognition of an existential need for humanity to embrace our interconnectedness to each other and all living things. Acknowledgement of these relationships provides a particular set of reasons *why* equity work matters. It also offers a particular set of approaches to *how* equity work is pursued. When social environments are understood as relational, the consideration or integration of equity within social systems and structures is also inherently relational. The SEA Framework centres a relational approach to thinking about and responding to issues of equity, across any range of contexts where it might be applied.

### Elements of the SEA Framework

**Fig. 1:**
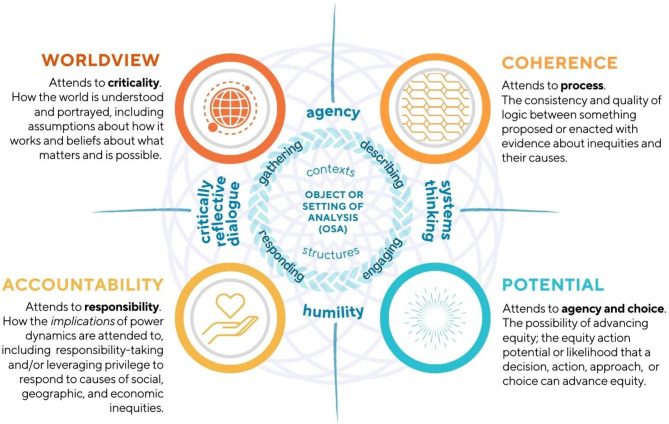
The Systematic Equity Action-Analysis (SEA) Framework

**Fig. 2:**
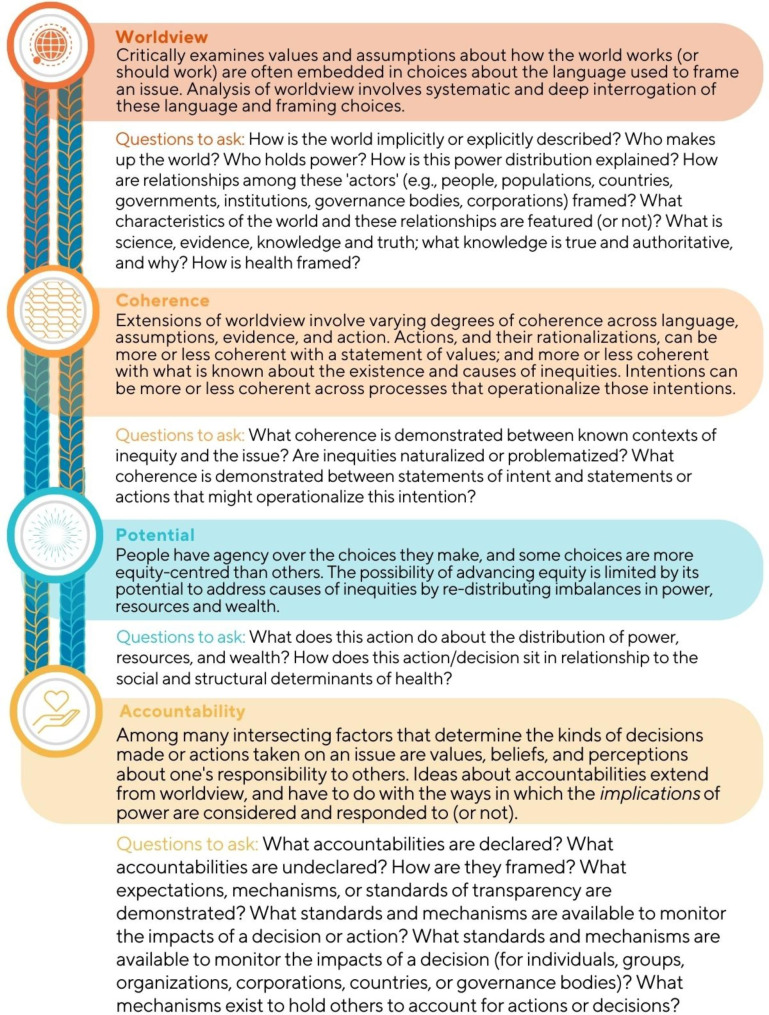
Sets of questions to ask across four elements of analysis

Inspired by Indigenous wisdom about the inextricable connection between knowledge and action, and the assumptions underlying both [28], the framework moves through four interwoven elements of analysis: *worldview*, *coherence*, *potential*, and *accountability*. As shown in Fig. 1, the four elements are relational, each flowing from the other, in both directions in interwoven, mutually reinforcing and influencing ways. Each element includes a particular set of analytical questions (detailed in Fig. 2) to apply to an *object or setting of analysis (OSA).* This *object* or *setting* sits at the centre of the framework, situated in sociopolitical contexts and structures. The object or setting of analysis can be anything where there is an opportunity to think about or act on issues of equity: a policy, a strategic plan, provision of health or other services, a course syllabus or readings list, a research proposal, a public announcement, an engagement plan, et cetera. Because equity issues are present in virtually all aspects of society, the kinds of ‘things’ the framework could be applied to is only limited by the imagination of its users. The framework is intended to directly implicate the agency and action of people whose daily work and spheres of influence are central to the construction of social systems and structures.

Moving outward in four directions from the centre, the SEA Framework features two sets of practices: positioned East to West are *critically reflective dialogue and systems thinking*; and North to South includes practices of *agency and humility*. Critically reflective dialogue, as a practice, considers (and aims to transform) power dynamics by consciously interrogating power inequities and social, cultural, and political contexts [15, 29]. Systems thinking invites attention on (often taken-for-granted) systems and structures, with deep appreciation for the circular nature of relationships within them. It is about becoming aware of often taken-for-granted norms, assumptions, and structures that collectively shape social systems [30]. Practices of agency and humility are future-facing. Practicing agency means resisting fear and apathy in favour of an *intentionally hopeful* belief that our individual and collective actions shape the systems we navigate and the futures we inherit [23, 25]. Practicing humility is about intentionally adopting a posture of openness and learning, seeking mutual understanding over asserting one’s own assumptions of knowing [31–33]. Together, these practices invite people to lean into curiosity with tenacity, optimism, and confidence in the possibility of transformative change.

*Relationality*, and the inherent interconnectedness of all things [34–36], including all elements of this framework, is conveyed by visual choices. A web-like backdrop of epitrochoids (intersecting curves, drawn by points drawn in circular rotation, extended from the outside of a fixed circle) extends from the centre circle to underlay the entire framework. This backdrop invites users to contemplate how each element exists in relationship with and to others. We choose to use circles, both at the centre, and to anchor each of the four elements of analysis, to convey the fluid, dynamic and non-linear nature of whatever OSA is being considered. Respect for the four directions, inspired and informed by many Indigenous authors, Elders, and leaders who teach us about ways of thinking and being in the world [37], show the relationships between *how* something is contemplated and *what* is contemplated. In Figs. 1 and 2, we use braids to emphasize the framework’s attention to *processes* that have the potential to bring together of many ways of knowing, and to demonstrate this work as an active effort to weave insights, considerations, and questions together toward something tangible, strong, and practical for use in equity work.

#### Worldview

Knowing and doing are inextricable, with assumptions about the world serving as a guide for both [28]. Depictions of the world and how it works involve choices and actions that are, in themselves, exertions of power. In this analytical element, attention is drawn to imaginative geographies [38] implicit or explicit in the OSA, unpacking assumptions about how the world works and how inequities are understood within it. Imaginative geographies, first described by Edward Said [39] in his discussion of the portrayals and framing of orientalism, are representations of space, and peoples inhabiting it, entangled with relations of power and a socio-spatial order.

These conceptualizations of space create divisions and relationships of other: of us/them, worthy/unworthy, developed/undeveloped, North/South. With respect to North-South relations, for example, Escobar argues these divisions reinforce “an extremely efficient apparatus for producing knowledge about, and the exercise of power over, the Third World” [40, 41]. Humans, by nature, make sense of the world and their experiences in it by constantly ascribing to (often contradictory) hegemonic assumptions that shape how we “explain situations, solve problems, and guide actions” [29]. Assessing worldview involves asking questions about how relationships in the world are discursively constructed, including the legitimization of positions or responses to issues of equity, leading to particular kinds of solutions [16]. It asks questions about intersectionality, including the construction of social relationships and positionality [42]. Crenshaw’s intersectionality lens challenges dominant group tendencies to structure issues of discrimination as separate or additive, and instead recognizes experiences as broader than general categories, attentive to multiplication or combined effects [42]. While there is ample evidence to the contrary, for example, health inequities are often portrayed as existing without known causes, as mysterious, or the simple bad luck of living in a poor country [43]. These pervasive and self-reinforcing, dominant epistemological and ideological norms play a role in the persistent failure of health research and health interventions to respond to inequities. The process of critical analysis about worldview, as portrayed in a text or policy, therefore, involves processes of recognizing and unpacking how often unquestioned assumptions shape what we believe we know, understand, and should do.

#### Coherence

Mismatches between causes and responses, or between words and actions, limit progress on equity. Two threads of analysis are embedded in exploring *coherence*: one between rhetoric and action, and another between action and evidence. At a time when most organizations and institutions are ripe with equity, diversity, and inclusion plans and intentions, there is a great deal of risk that bold statements are used to justify actions that merely reinforce unfair systems with a more diverse appearance [44, 45]. The tension between inclusion and tokenism is well explored in the literature, and less well resolved in practice. Assessing for coherence across aspirations and actions involves looking for the relational connections between a policy and the people who are involved in enacting and enforcing a policy. It explores alignment between words that sound equity-promoting, and the many layers of action required to operationalize these words.

Coherence between evidence and action applies questions about responsiveness to known causes of inequities. Solutions that miss root causes serve to maintain or distract attention away from the actual problem. For example, framing inequities in ways that effectively *naturalize* them, is incoherent with the evidence that points clearly to known causes [46]. This can happen when inequities are conceptualized as unavoidable characteristics of a ‘natural’ world, where they simply occur and therefore do not require explanation. By asking a series of questions about awareness and receptiveness to the known causes of inequities, we can explore how coherent something (a policy, a practice, a research methodology decision, etc.) is with the body of evidence about causes.

#### Potential

Action potential is a neuroscientific term that most health professionals and scientists learn about as the fundamental mechanism by which nerve cells communicate. If something inhibits a cell’s action potential, the nerve impulse is blocked and cannot respond. We choose this concept to invite people trained in basic, biomedical sciences to consider the action potential living in a proposed response to known inequities. In 2008, the World Health Organization’s Commission on Social Determinants of Health declared social and policy environments, and social injustices within them, as causes of health inequities [74]. Equity action potential, and its strength, is determined by choices. Assessing for *potential* involves asking questions about how a planned action is likely to affect the known causes of inequities, which can range from actions that reinforce and uphold inequities to those that disrupt root causes [46]. For example, in research, choices are made about how to frame a research problem or design a study; in health systems or municipal settings, choices are made about prioritizing policies; in intrapersonal communication, choices are made about how to respond to witnessing an injustice. All choices involve different kinds of potential, with some more permeable to advancing equity than others.

Greater equity action potential exists when people choose to critically examine the manifestations of structural, sociopolitical, and historical issues of power in their own settings. Health equity work is intensely complex and tied up in socio-economic, political, and historical conditions that are entrenched in global systems of power and hegemony [40, 41, 47]. Forces of political economy, for example, play a role in shaping these environments [2, 48, 49], wherein these forces differentially shape health and life trajectories along social status and power dynamics [50, 51]. Choosing to be more aware and aligned with the structural and systems-level forces that shape equity possibilities generates a greater likelihood that planned action will actually do *something* to advance equity.

#### Accountability

In 1998, Stan and Peggy Wilson of the Opaskwayak Cree Nation wrote an editorial about *relational accountability* in the (then named) Canadian Journal of Native Education [52]. They wrote as a team, which included a group of student researchers who had spent time in community on summer research projects. The group collectively reflected on their experienced dilemmas about how to report data, because their feelings of identity with and accountability to community challenged their training in research. They explored the concept of relational accountability, including works of other academics (e.g., Gergen), and found none approached the “depth of relational accountability that our students and other Indigenous scholars experience.” They connected their reflections to ceremony and prayer honouring *all my relations*. Much of what they grappled with was a deeply-rooted, heart-driven feeling of responsibility for their actions in relationship to others, and to the consequences of their actions.

Discussions of accountability span centuries, geographies, philosophies, and disciplines. Most health professionals and scientists receive training in biomedical or relational ethics. In global health, notions of care and accountability extend the accountability of nation-states to others in the world [53–55]. Yet, the dominance of neoliberal and capitalist ideology has pushed most notions of accountability toward effectiveness and efficiency [56–60]. In an age of populism, accountability to other humans, and recognition of our interconnectedness, are eroded [61, 62]. Aassertions of accountabilities tend to narrowly revolve around short-term cycles (e.g., annual budgets) or distracting public messaging and avoiding ‘bad’ publicity rather than accountability to each other and to upholding values like dignity, rights, or solidarity. Here, we invite analysis of how accountability is framed and enacted, with an unapologetic declaration that equity-advancing accountabilities emphasize collectivism and holding each other with care. Equity-advancing accountabilities include a commitment to honouring many ways of knowing [63]; and to thinking about the relationship between what we do now, today, and the futures of those yet to be born.

### Applying the SEA Framework

The SEA Framework offers a systematic approach to exploring equity integration in a variety of settings. For example, this framework might be used to examine a set of policy documents and observe the relationship between statements of intention and the actions that follow, with attention to the relational contexts that influence and determine both. In Tables 1 and 2, we offer four case examples of applications across the four elements of analysis. As we work to use this framework in more settings, future publications will offer more in-depth and nuanced examples of application. It could be used by groups external to an OSA by groups interested in systems that affect them, who engage in gathering publicly accessible and relevant documents (e.g., government policy statements); or by groups interested in transforming the system they are actively embedded in, who engage in gathering policy (or other) documents in their own settings and use critically reflective dialogue and systems thinking to unpack the ways in which equity is operationalized through their own work.

**Table 1:** Distinct SEA Framework Applications Across Four Elements of Analysis

	Worldview *How is the world understood and described? Who are the actors within it? How does power work within it?*	Coherence *How coherent is the logic between what is proposed and the evidence about inequities? How coherent is what is said with what is done?*	Potential *What impact will action have on known causes of inequities?*	Accountability *How is knowledge of power inequities responded to (particularly by those with more power)?*
**Example**	Case Example 1: Research project with questions about equity-centred practices in global health research funding in Canada.	Case Example 2: Deliberative dialogue study focused on learning from the role of vaccine nationalism in hindering more equity-responsive approaches to global health governance.	Case Example 3: Review and transformation of an undergraduate nursing curriculum for equity integration.	Case Example 4: Collaborative equity-framework building process with a municipal policy and planning team.
**Object or Setting of Analysis** **(OSA)**	Policies of Canadian funding agencies, with a focus on global health research.	Vaccine nationalism as a concept, using Canada’s policy trajectory as a case example to learn from; and the World Health Organization’s zero draft for a proposed pandemic instrument or treaty.	First-year curriculum in an undergraduate nursing program, including a series of documents relevant to the design and delivery of an undergraduate nursing program (e.g., course descriptions and learning objectives in the academic calendar; course syllabi; student testimonies).	A municipal policy and planning team, including a series of documents relevant to their work (e.g., Official Community Plan, municipal procedures, meeting notes and reports).
**People Involved**	This retrospective project was led by people who engage in global health research and apply for funding from funding agencies (but were not employees of funding agencies).	This prospective project was led by a team of people who championed mobilization of the global-public health community over the course of the pandemic, issuing direct calls for equity- and evidence-informed action from the Government of Canada with respect to its foreign policy.	This planned action project was facilitated by a School of Nursing Equity Committee (leadership, research faculty, teaching faculty, and students) and invited instructors who design and deliver first-year courses to students in the program into a learning community.	This planned action project was facilitated by equity scholars and practitioners, with policy and planning staff and management, and collaboration from managers in other teams that work closely with them.
**Process Highlights**	*Gathering* activities involved harvesting a series of publicly available documents. For example, source documents for the Canadian Institutes of Health Research (CIHR) included the CIHR Strategic Plan 2021–2031; Tri-Council Guide on Financial Administration; a selection of current global health-relevant funding calls (open project grants, priority calls); and the CIHR Peer Review Manual. *Describing* activities involved Descriptive analysis, using qualitative content analysis and preliminary coding framework, followed by critical discursive analysis guided by Brisbois and Plamondon (16), and Johnstone (73). Key informant interviews with internal funding agency partners were invited, as part of a *listening* activity.	*Gathering* activities involved a series of knowledge synthesis, used to inform a series of deliberative dialogues amongst people with expertise in global governance, ethics, and equity. The research team applied to be a recognized stakeholder to provide review and provide feedback on the zero draft of a proposed ‘pandemic instrument’ or treaty, prepared by the Intragovernmental Negotiating Body (INB) of the World Health Organization (WHO). *Describing & Listening* Deliberative dialogue-informed process of equity analysis, point-by-point on the draft instrument (September 2022), followed by participation in a series of consultation sessions hosted by the INB (September and October 2022). Particular attention was directed at action-analysis for coherence between stated intentions and proposed interventions. *Responding* involved submission of an in-depth equity analysis of the zero draft, with specific recommendations for the INB to consider in the December meetings.	*Gathering-Describing-Listening-Responding* activities were integrated throughout a series of critically reflective dialogue workshops. With a commitment to learning-from-doing, instructors were invited to work collaboratively with equity scholars to complete an equity action-analysis. Teams began with capacity-building workshops build shared goals, clarify assumptions, and learn about the equity experiences of students. Collaborative document analysis was completed in a dialogic setting, including leadership from the School of Nursing, equity scholars, students, and instructors. Workshops were offered over the course of a year, inviting instructors to learn from each other about cultivating equity-responsive learning environments. An online collaborative learning platform was established to share resources. Changes were made to course descriptions and syllabi. An evaluation was completed, inviting students to reflect on their experiences in the revised courses.	*Gathering-Describing-Listening-Responding* activities were integrated throughout a 12-month process of capacity building, using a series of half-day workshops in a dialogic setting including leadership from several teams across a municipality, equity scholars; a complementary series of ‘design labs’ and a set of knowledge mobilization activities.
**Findings**	Across multiple funding agencies, discourse analysis revealed a strong rhetoric of equity discursively constructed the world as a place of inequities, without explanation, context, or evidence to situate those inequities as having known causes. Biological determinants of health were afforded the same importance as social, cultural, environmental, and structural determinants (and no evidence or theory was cited). The legitimacy of research as a means of advancing equity was frequently assumed, without justification. Research is implicitly described as benevolent and important for accelerating health equity, with little to no explanation for how this will happen, what specific role research will play, or what the relationship is between inequities, determinants of health, and research. Policies uniformly did not draw attention to the role of mitigating power or resource inequities in the context of research. Policies suggest Canada plays an important role in helping other countries with their health, reinforcing dominant, western discourses of generosity, benevolence while ignoring the socio-historical and political roots and current conditions which provide Canada global privilege.	The proposed instrument, intended to operationalize equity more effectively in the context of future pandemics, does not name causes of inequities, or refer readers to reliable sources for explanation. Risks promoting and/or validating research that mismatches cause and response. While the proposed instrument offers strong statements about acting on the causes of inequity it fails to connect these with coherent actions. Since the draft pandemic treaty “shall not affect the rights and obligations of any Party deriving from other existing international instruments” it is unclear how coherent this instrument may be with other international treaties and laws.	Equity review of language in the course descriptions and learning objectives. Recognizing the role nursing curriculum plays in shaping the future of the profession, this project sought to directly transform curriculum design and delivery in ways that were more equity-promoting. The project used the ‘disrupt-distract’ tool (46) to support dialogue about the equity choices embedded in designing and delivering courses. Critically reflective dialogue in a course-by-course process, using course syllabus alongside reflection on how courses are equity-responsive in both structure/design and in the cultivation of learning environments.	Equity practices and considerations were examined across the routine processes and aspects of municipal work. While elements of worldview, coherence and potential were also all addressed in this project, accountability became a central focus of the work. The process led to the creation of the *Ripples of Change: Integrating equity thinking in municipal work for better futures.* The document provides people with foundational background for understanding the role of equity in municipal work, drawing attention to the ways in which individuals, teams, and the municipality all collectively contribute to shaping equity possibilities within a community. The framework invites users to recognize their own role in equity work, and to commit to engaging with each other, building capacity, listening and learning, and making time and space to think about (and make) equity-centred choices. Importantly, the framework invites integration of equity questions across all aspects of day-to-day, policy, operational, and strategic work in municipal settings.
**Next Steps**	In Spring 2023, the research team will host a series of dialogue-based workshops with a diversity of people connected to funding agencies (peer reviewers, researchers, students, community members, agency staff) to consider implications and invite responsiveness.	Direct offers for support in identifying strategies for operationalizing equity through the treaty were made to the INB Co-Chairs. Further to efforts to equip INB members with vocabulary and strategies to operationalize equity, the process is informing a second round of deliberative dialogues that will invite consensus-building around obligations to others (beyond individual or nationalistic obligations) in the context of inherently global health issues. After the instrument is finalized, WHO Member State ratification is anticipated in 2024.	Efforts are now underway to extend the process of review to subsequent years of the program, engaging a growing group of instructors in the process. A student advisory group is being established to create more opportunities for student voices and experiences in shaping equity-promoting design and delivery of curriculum.	Efforts are underway to explore how to operationalize the framework. In October 2022, municipal elections resulted in the formation of a new City Council (including a new mayor). Changes in leadership within the municipality will play a role in determining what next steps might be possible. Equity scholars supporting the project continue to offer their support, leveraging their academic freedom to speak to issues of equity in ways that people within the administrative bureaucracy may not be able to do.

**Table 2:** Four elements of analysis, applied across a case example of a deliberative dialogue study on vaccine nationalism

Example The Solidarity for Vaccine Equity (SOLVE) study uses deliberative dialogue to learn from the role of vaccine nationalism in hindering more equity-responsive approaches to global health governance (https://www.solve-study.ca/).		
SEA Framework Element	Description of analytic content and processes	How analysis was accompanied by action
**Worldview** *How is the world understood and described? Who are the actors within it? How does power work within it?*	Study analyzed the worldview as portrayed by the Government of Canada, through an in-depth analysis of publicly available policy documents. Particular narratives of Canada in the world were identified, and situated in the context of policy actions proposed and taken. Observations were made during global governance proceedings on the worldviews advanced by different actors involved in the World Health Organization’s Intergovernmental Negotiating Body (WHO-INB) in the elaboration of a new pandemic instrument/treaty/accord (known as WHO CA+).	Document analysis unfolded alongside scholar activism, with the SOLVE Study research team gaining designated stakeholder status with the WHO-INB. Results emerging from policy analysis and deliberative dialogue were used to guide equity- and evidence-informed contributions to the INB proceedings for the development of the conceptual zero draft and continued through to offering interventions in proceedings after the release of the zero draft of the proposed instrument or treaty/accord (titular language for which is yet to be negotiated).
**Coherence** *How coherent is the logic between what is proposed and the evidence about inequities? How coherent is what is said with what is done?*	Alignment between rhetorical statements made by the Government of Canada and actual policy directions were mapped out as part of the policy analysis. Coherence in Canada’s policy trajectory was further assessed by examining the ways in which pandemic-related inequities were portrayed. This analysis was extended to iterative drafts of the proposed WHO CA+, evolving through the WHO-INB proceedings.	Demonstrations of incoherence between a rhetoric of equity and Canada’s actual policy actions were highlighted in a number of different settings, including national and international forums that included policy makers (e.g., lead author Plamondon delivered a keynote presentation entitled “*Holding onto equity in a political ecology of nationalism, capitalism, and neocolonialism*” at the March 2023 Government of Canada Pandemic Instrument Partner & Stakeholder Engagement Forum). Line-by-line review of drafts of the pandemic treaty identified points of incoherence and provided specific recommendations on how to promote greater coherence. These reviews were submitted to the WHO-INB for consideration in successive iterations of the proposed WHO CA + and guided interventions delivered at proceedings of the WHO-INB (see: https://www.solve-study.ca/reports-publications). Research team members attended all four sessions for the WHO CA + informal, focused consultations (IFC) Sept-Oct 2022 and provided real-time comments to highlight implications for equity coherence and equity potential.
**Potential** *What impact will action have on known causes of inequities?*	As with coherence, Canada’s policy actions were analyzed in the context of the evidence on known causes of inequities for their possible impacts. This analysis involved monitoring of relevant data and literature as it became available (e.g., global distribution of vaccines; modelling on deaths averted due to access to vaccines). Given the WHO CA + emphasis on the new instrument as centering equity as a driving principle and primary outcome, we extended the same analysis of possible equity impacts to iterative drafts.	
**Accountability** *How is knowledge of power inequities responded to (particularly by those with more power)?*	Accountability, in this study, is a central question guiding deliberative dialogues. For each gathering, we posed (and continue to pose) questions about when collective obligations to humanity supersede obligations to protect state-based rights and interests. All participants exposed to these dialogues receive documents naming the pre-existing contexts of inequity in which the pandemic unfolded and its direct impacts on amplifying inequities. These materials summarize the evidence that demonstrates prioritization of profits and lives for those living high-income countries. The study invites people to consider their own spheres of influence as part of the dialogic process.	Accountability, as a concept, is the guiding star for this study and all analytic and action-focused activities as described throughout table (e.g., engagement with stakeholders in deliberative dialogues, participation in the WHO INB proceedings including contributions to iterative drafts of WHO CA+) are done with the ultimate aim to ensure those in positions of power (e.g., Government of Canada, WHO INB, individuals within their own spheres of influence) act with accountability to those who have less power during a pandemic.

Essential to its application is a mix of capacities and perspectives. In our experience, equity analysis is best done by teams that reflect a diversity of perspectives, and in processes that involve active consultation with individuals, groups, or communities most affected by the policies, practices (or other items) of interest. This may involve people who are in positions of decision making and authority over shaping a particular policy or practice climate; but it most importantly involves those whose voices are often over-shadowed, while simultaneously bearing greater burdens or impacts within a particular policy or practice climate. Their tacit knowledges and expertise are essential to identifying the most relevant and important content for analysis. If, for example, a team is working with a community group, then voices of people most affected by the policies in question should be actively involved in all phases of analysis: gathering, describing, engaging, and responding.

#### Before using this framework

Redistributing power is a central problem of advancing equity [64]. Those who wish to use this framework require foundational training or experience that equips them with the capacity for reflexivity and critical engagement in recognizing, understanding, and mitigating issues of power [65]. This prerequisite can present a challenge because of the persistent dissonance between evidence and action in the health sciences that share broad goals of reducing inequities and promoting equity, such as in global and public health. Fortunately, increasing interest in responding to this gap means there are many resources available to support strengthened capacity, including literature and resources to support deepened understanding of the role of privilege in relationship to issues of equity [17], and for engaging in critical analysis generally [66], or exploration of different imaginative geographies of the world [38]. There are excellent resources to support reflexivity and the inter-personal work of learning about anti-racism, cultural safety, and cultural humility [33, 67–69]. An incredible diversity of resources is available to support both individual and collective engagement in reflexive learning, ranging from training in cultural humility to workbooks, videos, articles, and online communities. Important among these works are the arts [70, 71], which serve as an evocative pathway to consciousness raising, re-presenting and questioning often taken-for-granted assumptions. In our experience, this work is difficult and mind-bending at times—it is best to move through the following phases and elements of analysis, engaging in these sets of practices with honesty and laughter—it makes the intensity of this work much lighter!

#### Gathering

Application of the SEA Framework begins by gathering people and information. Gathering people can be considered a process of building a place of welcome and invitation into the work of systematic equity action analysis. In this phase, people with a shared goal of advancing equity come together in dialogue to articulate the reasons why they want to work together toward equity and identify the focus of their equity work. Importantly, the process of gathering must be authentic. This requires an active commitment to a practice of inclusion, resisting and countering tendencies to token or exploitive approaches that may serve to reproduce rather than transform power imbalances [13, 64]. While there is shared responsibility, the work implicates differentiated responsibilities among those who navigate systems from different positions of power or unearned advantage/disadvantage [30]. Systems transformation requires active listening, participation, and commitment from everyone who navigates the system.

This might be a team interested in integrating equity in their workplace, or it might be a graduate student who is weaving equity into their thesis work. This initial team, in the early stages of gathering, collectively identifies the *OSA*. A good equity practice, at this point in the process, is to be observant and curious about who is at the table, who is engaged, and how. As the process unfolds, there may be good reasons to extend invitations to others—always asking equity-centred questions about how this group of people can become more aware of the equity implications of their work. Gathering people can continue throughout systematic equity analysis, as different phases might open opportunities or need for more perspectives.

The group can then work together to create a strategy for identifying and gathering the data and designing a process to support analysis. The *data* gathered to support analysis will vary depending on the nature and social location of OSA (some examples are offered below). If, for example, the framework is being used to assess a policy, a group’s strategy might include gathering documents, websites, public announcements, and other materials that serve to: (a) articulate the policy scope and details; and (b) describe sociopolitical contexts and structures of relevance. This can include harvesting grey or peer-reviewed literature, exploring population-level data, and identifying texts, video, news releases, or other sources of information that are essential to understanding the OSA. At this stage of analysis, we find it helpful to create a data extraction table (e.g., in MS Excel) that tracks the items that make up the data set. Similar to how one might start systematically detailing the descriptive content for a literature review, we harvest descriptive data available on these documents (e.g., date, author(s), web links, purpose statements, et cetera). Together, this data gathering process serves to create a beginning data set for analysis and dialogue.

#### Describing

With a beginning set of materials and group of people gathered, efforts to engage in listening and describing can begin. In this phase, teams involved in the analysis listen to each other and others about why doing this equity work matters. With a shared purpose and drive behind the work, teams can then work together to define a process for interacting with and coding the data. It can be good to start this interaction by deeply describing and understanding the OSA, using secondary sources of data, literature, oral histories, story-telling, context-mapping, or whatever detailing approach is deemed most suitable by the people most affected by the analysis. Written or visual data can then be analyzed for worldview, coherence, potential, and accountability. Starting with Worldview is important, because the others flow from whatever ways the OSA serves to construct the world and how it functions. There may be limits to what can be assessed for the other three elements of analysis, but these limits are—themselves—important to identify. These limitations inform questions to explore in the next phase (engaging).

People with experience in qualitative content analysis or discourse analysis are well suited to the time-intensive work of examining and coding data sets. Practically, this phase is best done with the support of some kind of organizing software (e.g., NVivo, Mendeley, MS Excel). Though some of the questions for each element may not be fully answer-able in every possible text, silences or absence of attention to elements named in the framework can also offer helpful insights. When questions are un-answerable from observation of the team, they can be flagged for consideration in the next phase.

#### Engaging

Dialogue is essential to all phases of the SEA Framework, but is particularly central to making sense of the implications of preliminary analysis. Making sense of what is discovered through the *gathering* and *describing* work of the SEA Framework requires engaging with broadly representative groups of perspectives and experiences. Attentiveness to issues of representation and inclusion is a central principle of equity-centred engagement in using and doing research [31]. Teams can and should be intentional in asking themselves whose voices might be missing, and designing strategies to ensure those voices are meaningfully included. By engaging broadly, a deeper and more nuanced analysis can open. Analysis will be more nuanced and richer when people who understand context, practice, spheres of influence in depth are part of the process. It is important to engage people both affected by the OSA (a policy, for example), and people who are in positions of authority over that same object. This engagement brings perspective that analysis of texts alone cannot.

With data coded and organized around the four elements of the framework, *engaging* involves dialogue with relevant, affected people and groups—asking questions of interpretation. In this phase, dialogue serves to clarify possible implications of findings, asking ‘*what does this mean?’* and ‘*why does it matter?’* This phase extends iterations of understanding, working together with groups to identify where equity is integrated (or not). If the framework is being used with groups who have authority or agency to participate in shaping the policy or practice climate of interest, this phase of analysis invites them to consider where they might find opportunity to integrate equity considerations more fulsomely. Application may be possible from a distant, outside-observational gaze; but dialogue with people affected by the object under analysis, particularly that invites multiple perspectives and disciplines, will always lead to a more fulsome consideration of that ‘something’. The more pluralistic and learning-focused, the better.

#### Responding

As groups move through using and applying the SEA Framework, they generate new understanding around assumptions and systems that inhibit equity, and gain insight into the equity implications of the OSA. The final phase of analysis is about making choices on how to respond. The nature and direction of the response will be shaped by who is doing the analysis, and on what object. Regardless of any individual or group’s position or authority, *responding* relies on recognizing and leveraging their spheres of influence. Responding might, for instance, involve strategically working to better align vision statements with operational policies, or integrating equity conversations into routine meeting agendas. Our concise description is not intended to minimize the importance of this work: responding is the linchpin of advancing equity. This is the moment where those involved make choices about how the work informs their own daily practices, including thinking habits and routine ways of working. It is a moment of planned action, embracing the spheres of influence available to those using the framework in an intentional, deliberate way.

## Flexibilities: how to nuance and adapt

Several different applications of the SEA Framework informed its evolution and refinement, including a variety of settings and contexts. Some examples include application in a funding policy analysis (Case Example 1), where document review of Canadian funding agencies’ strategic and operational policies was complemented by key-informant interviews. The study included document review for international comparators, asking a consistent set of equity-centred questions to illuminate the degree to which equity considerations are integrated across these kinds of policies. Using an engaged process of critically reflective dialogue with a diverse audience of people with expertise and interest in the policy implications of vaccine nationalism, another study examines collective obligations and issues of solidarity in global governance over the course of the COVID-19 pandemic (Case Example 2, see Table 2 for in-depth presentation of this example). Another setting involved insights from efforts to decolonize nursing curriculum (Case Example 3), using an 18-month process of deliberative dialogue to offer curriculum leaders and instructors opportunities to build collective capacity to critically reflective evaluation of strategic policies (course descriptions, goals, learning outcomes), operational policies (course syllabi, resources/readings, assignments), and practice elements of teaching and learning. In another project, emergent elements of the SEA Framework informed a process of systems change at a municipal government through a one-year process of engagement alongside policy and practice analysis and document review (Case Example 4). In this example, the focus was on using systematically unpacking current policy as a mechanism for building capacity for integrating equity considerations in future policy, planning, and practice. In each of these examples, a team of people with varying degrees of experience (us, as authors, among them) worked in collaboration with external teams to advance a shared equity goal. Equity work is messy and complex with no fixed end-point. We offer promising highlights for these four case examples, recognizing the work for each is ongoing and requires anticipating and responding to unintended consequences as we stumble imperfectly, ungracefully, and humbly through this work.

### Implications and future considerations

Critical activists and scholars continue to call for transformation that aligns with and amplifies rising public debate and social movements seeking to delegitimize western, colonial, and white-centric systems of knowledge, power, and oppression. Increasing attention on advancing equity follows a well-established (and long) history of social movements and public outcry over the persistence of injustices and systemic discrimination, most recently including the Occupy, MeToo, and Black Lives Matter movements [72]. We add to this scholarship a framework for analysis *and* planned action that balances critical thinking with practice.

At a time of ubiquitous equity rhetoric, any framework purporting to support equity work is at risk of disingenuous or superficial use. While we recognize no one approach can offer a panacea to inequities, the SEA framework offers a strategic response to long-observed stagnation in our own fields of practice [12]. Despite our efforts to challenge reductionism and linearity, the SEA Framework emerged from a particular set of experiences, theories, and literatures. Like any framework might, its application must be undertaken with awareness of the always-present risks of overlooking or minimizing aspects of this complex work.

With its distinct focus on a practice of recruiting power-holders to dismantle systems that hold their unearned advantages in place, we hope this framework provides a means of gaining traction to advance equity more quickly and meaningfully. It is both critically reflective and future-facing, creating space for imagination and hope that can serve as a platform for collaboration and action-planning. This framework may require updates and nuance, adjustments and expansion—with adaptations to different settings, and continued efforts to demonstrate what difference it is making.

## Conclusion

While efforts to advance equity in the health field span decades, research norms and patterns within the health disciplines tend to be poorly aligned with their aspirational ideals. In its structure and process, with a focus on stimulating critical reflection and systems thinking among people who enact transformative work from within the systems they themselves are positioned, leveraging their own agency to act, the SEA Framework moves equity and power analysis beyond a *thought exercise*. Returning to the late, incredible bell hooks, we are inspired by her compellingly radical calls to act on inequities in society through love, compassion, and hope. We invite readers to use this framework, and share your experiences.

## Data Availability

All datasets related to the study are included in this published article. For inquiries regarding our critically reflective process, or for direction to publications on various research projects our group has been involved, may be made to katrina.plamondon@ubc.ca.

## Abbreviations

OSA: Object or Setting of Analysis
SEA: Systematic Equity Action-Analysis
